# *Campylobacter jejuni* from Slaughter Age Broiler Chickens: Genetic Characterization, Virulence, and Antimicrobial Resistance Genes

**DOI:** 10.1155/2022/1713213

**Published:** 2022-05-19

**Authors:** Tsepo Ramatla, Kealeboga Mileng, Rendani Ndou, Mpho Tawana, Lehlohonolo Mofokeng, Michelo Syakalima, Kgaugelo E. Lekota, Oriel Thekisoe

**Affiliations:** ^1^Department of Animal Health, School of Agriculture, North-West University, Private Bag X2046, Mmabatho 2735, South Africa; ^2^Unit for Environmental Sciences and Management, North-West University, Private Bag X6001, Potchefstroom 2531, South Africa; ^3^University of Zambia, School of Veterinary Medicine, Department of Disease Control, P.O. Box 32379, Lusaka, Zambia

## Abstract

*Campylobacter jejuni* is a major cause of food-borne human gastroenteritis worldwide and is designated as a high priority antimicrobial-resistant pathogen by the World Health Organization (WHO). In this study, a total of 26 *C. jejuni* isolates from broiler chickens were screened for the presence of virulence and antimicrobial resistance genes by PCR. As a result, the study detected 11/26 (42.3%), 9/26 (34.6%), 8/26 (30.8%), 7/26 (26.9%), 6/26 (23.1%), and 6/26 (23.1%) of *cdtC, pldA, cdtB, cdtA, cadF,* and *ciaB* virulence genes, respectively, with seven of the isolates carrying more than two virulence genes. The majority of the isolates *n* = 25 (96.1%) were resistant to nalidixic acid, followed by *n* = 21 (80.7%), *n* = 22 (84.6%), and *n* = 5 (19.2%) for tetracycline, erythromycin, and ciprofloxacin, respectively. Most isolates were harboring *cat*I (*n* = 16; 84.2%), *cat*II (*n* = 15; 78.9%), *cat*III (*n* = 10; 52.6%), *cat*IV (*n* = 2; 10.5%), *flo*R (*n* = 10; 52.6%), *erm*B (*n* = 14; 73.7%), *tet*O (*n* = 13; 68.4%), *tet*A (*n* = 9; 47.4%), *mcr-4* (*n* = 8; 42.1%), and *amp*C (*n* = 2; 10.5%). Meanwhile, *mcr-1, mcr-2, mcr-3, mcr-5, tet*(X), *tet*(P), and *tet*(W) genes were not detected in all isolates. Class I and Class II integrons were detected in 92.3% (*n* = 24) and 65.4% (*n* = 17) isolates, respectively. About 31% (8 of the 26 isolates) isolates were carrying more than two resistance genes. According to our knowledge, this is the first study to detect class II integrons in *Campylobacter* spp. (*C. jejuni*). The high prevalence of *cdtA*, *cdtB*, *cdtC, cadF*, *pldA*, and *ciaB* genes and antibiotic resistance genes in *C. jejuni* in this study indicates the pathogenic potential of these isolates. Majority of the isolates demonstrated resistance to nalidixic acid, tetracycline (*tet*), and erythromycin (*erm*B), which are the drugs of choice for treating *Campylobacter* infections. Therefore, these findings highlight the importance of implementing an efficient strategy to control *Campylobacter* in chickens and to reduce antimicrobial use in the poultry industry, which will help to prevent the spread of infections to humans.

## 1. Introduction

Poultry meat is an important source of protein and one of the most consumed meat sources in South Africa [1]. To date, about 2.152 million tonnes of poultry meat are consumed in South Africa per year [2]. Despite chickens being considered the main source of protein, they are also responsible for about 80% of human cases of food-borne and zoonotic diseases [3]. Poultry is considered as the main reservoir of many bacterial pathogens including *Campylobacter* [4]. A number of chicken-borne *Campylobacter* species of zoonotic importance include *C*. *ureolyticus*, *C*. *concisus*, *C*. *mucosalis*, *C*. *jejuni*, *C*. *hyointestinalis*, *C*. *insulaenigrae*, *C*. *sputorum*, *C*. *helveticus*, *C*. *lari*, *C*. *fetus*, *C*. *coli*, *C*. *upsaliensis*, and *C*. *rectus* [5]. Of these, *C. jejuni*, *C. lari*, and *C. coli* are documented as the main contributors to food-borne diseases such as campylobacteriosis in humans [3, 6, 7].

In humans, *Campylobacter* infections are usually self-limiting, although bacteraemia is more common among the elderly, immunocompromised people, and children [8]. In comparison with other enteric bacteria, *Campylobacter* has multiple cell surface layers expressing virulent factors that are responsible for its high prevalence and pathogenicity [9]. Motility, toxin production, mucus colonization, attachment, and translocation are all virulence mechanisms used by *Campylobacter* to cause disease [10]. The antibiotics used for treating *Campylobacter* infections will usually target these mechanisms of virulence in order to be efficacious.

Different antibiotics such as erythromycin, amoxicillin, azithromycin, clarithromycin, tetracycline, and ciprofloxacin have been used for treating campylobacteriosis [11, 12]. In animals, some of these antibiotics are used as additives to improve the growth rate and feed intake ratio [13, 14]. As a result, the misuse of these antibiotics as additives can lead to antibiotic residues on animal products and the environment as well as the development of antibiotic resistance [1, 11]. Antibiotic resistance is a global health issue that involves the transfer of bacteria and genes between humans and animals [15]. A number of genes which confer resistance to antibiotics in *Campylobacter* have been determined by previous studies [2, 6, 11]

Tetracycline is exported from the cell via membrane-bound efflux proteins encoded by the efflux genes *tet*A and *tet*B [16, 17]. Tetracycline resistance is caused by the *tet*O gene, and it produces a ribosome-protective protein [16]. A ribosomal methylase encoded by *erm*B is one of the *Campylobacter* mechanisms that confer resistance to macrolides [16, 17]. The C257T mutation in the gyrase gene (*gyr*A) in *Campylobacter* is the most common mechanism creating quinolone and fluoroquinolone resistance [18]. In *C. jejuni*, the *gyr*A gene region contains Thr86Ala, which is responsible for high levels of nalidixic acid resistance and low levels of ciprofloxacin resistance [15, 19]. The Thr86Ile amino acid alteration in the QRDR of *gyr*A is seen in most ciprofloxacin-resistant *Campylobacter* spp., especially *C. jejuni* strains [20]. Antimicrobial resistance genes such as *erm*(B), *aad*E, b*laOXA*_−61_, and aphA-3 have also been linked to multidrug resistance in *Campylobacter* strains [3].

Integrons, in particular, play a key role in the acquisition and spread of antibiotic resistance [21, 22]. There are five classes, but only two classes, i.e., I and II are the most important [23]. Class I and II integrons are frequently associated with the Tn7 transposon family [24, 25]. Gram-negative bacteria have a wide range of class I integrons, which are transferred by Tn402 [21]. Dihydroflavonol-4-reductase (*dfr*), sulfonamide (*sul*1), broad-spectrum-lactamase, quaternary ammonium compound disinfectants (*qacE*1), and aminoglycoside-modifying enzymes (AMEs) are all encoded by antimicrobial resistance gene cassettes found in class I integrons [21]. However, the int gene in class II is less active, it can carry unusual cassettes that encode the lipoprotein signal peptidase [21, 26], and it has Dfr1, sul1, and aadA1 gene cassettes [21]. Even though integrons have been detected from class I in *Campylobacter* [27], neither class II nor III have been detected in *Campylobacter* spp. [27]. From the study conducted by van Essen-Zandbergen et al. [28] in the Netherlands in broilers, none of the *Campylobacter* isolates carried the integrons (class I, II, and III). Hence, the aim of this study was to investigate the presence of the virulence genes profile. Class I and II integrons and antimicrobial resistance genes in *C. jejuni* isolates recovered from the faeces of slaughter-age broiler chickens in the North West province, South Africa.

## 2. Materials and Methods

### 2.1. Identification of *Campylobacter*

26 *Campylobacter jejuni* strains from our previous study were used [4]. In brief, *C. jejuni* was isolated from faecal samples, and the genomic DNA was extracted following Zymo Research Fungal/Bacterial DNA kit instructions (Zymo Research Corp., CA, USA). The DNA concentration was quantified using a NanoDrop spectrophotometer [29]. Conventional PCR was used to detect the *Campylobacter* spp. in the chicken faeces using universal 16S *rRNA Campylobacter* spp. All the PCR products were sequenced at Inqaba Biotechnical Industries (Pty) Ltd., Pretoria, South Africa, and sequence identity was determined using the nucleotide Basic Local Alignment Search Tool (BLASTn) (https://blast.ncbi.nlm.nih.gov/Blast.cg). The nucleotide sequences were deposited in the GenBank database and assigned with accession numbers (MZ209102 − MZ209127) available at https://www.ncbi.nlm.nih.gov/nucleotide.

### 2.2. Antimicrobial Resistance (AMR) Profile

Antibacterial susceptibility screening to ciprofloxacin (5 g), nalidixic acid (30 g), erythromycin (15 g), and tetracycline (30 g) (Davies Diagnostics, Johannesburg, South Africa) was conducted based on the World Health Organization (WHO) Advisory Group on Integrated Surveillance of Antimicrobial Resistance guidelines [30] on food-borne bacteria. The Kirby–Bauer disc diffusion method was used, and the results were interpreted according to the Clinical and Laboratory Standards Institute (CLSI) [4]. Following CLSI recommendations, antibacterial susceptibility testing was performed on Muller–Hinton (MH) agar (LAB M, Neogen Company) supplemented with 10% sheep blood. The zones of inhibition detected around each antibiotic disc in millimeters were used to calculate antibiotic susceptibility. Standard reference strains of *Staphylococcus aureus* (ATCC® 29213) and *Campylobacter jejuni* ATCC (33560) were used as controls.

### 2.3. Detection of Antibiotic Resistance Genes

The presence or absence of chloramphenicol (*cat*I, *cat*II, *cat*III, *cat*IV, and *flo*R), erythromycin (*erm*B), tetracycline (*tet*(A), *tet*(O), *tet*(X), *tet*(P), and *tet*(W)), colistin (*mcr-1, mcr-2, mcr-3, mcr-*4, and *mcr-5*), and ampicillin (*Amp*C) resistance genes, including two classes of integrons (*Int*) (class I and II), was determined in *Campylobacter jejuni* isolates using the qualitative PCR technique. All the primers were obtained from Inqaba Biotechnical Industries (Pty) Ltd., Pretoria, South Africa. Each PCR reaction included a total reaction of 25 *μ*L containing 12.5 *μ*L of a 2X DreamTag Green Master Mix (0.4 mM dATP, 0.4 mM dCTP, 0.4 mM dGTP, 0.4 mM dTTP, 4 mM MgCl_2,_ and loading buffer) (ThermoFisher Scientific, South Africa), 8.5 *μ*L of nuclease-free water, 2.0 *μ*L of the template DNA, and 1.0 *μ*L of each oligonucleotide primer. PCR reactions were performed using the ProFlex PCR System (Applied Biosystems, USA). Amplified PCR products were resolved on a 1.5% (w/v) agarose gel in a 40 mM Tris (Sigma Aldrich, US), 1X TAE buffer (20 mM acetic acid (Merck, US), and 1 mM EDTA (Merck, South Africa) at pH 8.0), stained with 0.001 *μ*g/mL ethidium bromide, and visualized under ultraviolet (UV) light using the ENDURO GDS Gel Documentation System (Labnet International Inc., US). A molecular weight marker, 100 bp ladder (PROMEGA, Madison, WI, USA), was used to determine the size of the PCR product. A ChemiDoc Imaging System (Bio-Rad ChemiDoc^TM^ MP Imaging System, UK) was used to capture the images using Gene Snap software, version 6.0022. The primers and PCR cycling conditions used in the study are shown in Table 1.

### 2.4. Determination of Virulence Genes

Six virulence genes; Cytolethal distending toxin subunits; *cdt*A, *cdt*B, and *cdt*C, *Campylobacter* adhesion to fibronectin protein (CadF), phospholipase A (*pld*A), and *Campylobacter* invasion antigen B (*cia*B) were screened from *Campylobacter jejuni* isolates. The primers and PCR cycling conditions were obtained from the previously published articles [10, 38, 39] and are shown in Table 2. The 25 *μ*L total reaction consisted of 12.5 *μ*L of a 2X DreamTag Green Master Mix (0.4 mM dATP, 0.4 mM dCTP, 0.4 mM dGTP, and 0.4 mM dTTP, 4 Mm MgCl_2_, and loading buffer), 8.5 *μ*L of nuclease-free water, 2.0 *μ*L of template DNA, and 1.0 *μ*L of each oligonucleotide primer. PCR reactions were performed using the ProFlex PCR System (Applied Biosystems, USA). Amplified PCR products were resolved on a 1% (w/v) agarose gel in a 40 mM Tris (Sigma Aldrich, US), 1X TAE buffer (20 mM acetic acid (Merck, US), and 1 mM EDTA (Merck, South Africa) at pH 8.0), stained with 0.001 *μ*g/mL ethidium bromide and visualized under ultraviolet (UV) light using the ENDURO GDS Gel Documentation System (Labnet International Inc., US).

### 2.5. Data Analysis

Statistical analysis was carried out using Microsoft Excel 2016 (Microsoft Corporation, Redmond, DC, USA) and Statistical Package for the Social Sciences v. 26 (IBM Corporation, Armonk, NY, USA). The sequenced 16S rRNA gene of the 26 isolates was compared to nucleotide sequences available in GenBank. The closest representative sequences of *Campylobacter* spp. strains were retrieved and aligned with the sequenced isolates using ClustalW program for phylogenetic analysis. The phylogenetic tree was constructed in the MEGAX package using the maximum likelihood method and Kimura 2-parameter model [40] with a bootstrap analysis of 1000 replicates [41].

## 3. Results

### 3.1. Molecular Detection of Virulence Genes

**Figure 1:**
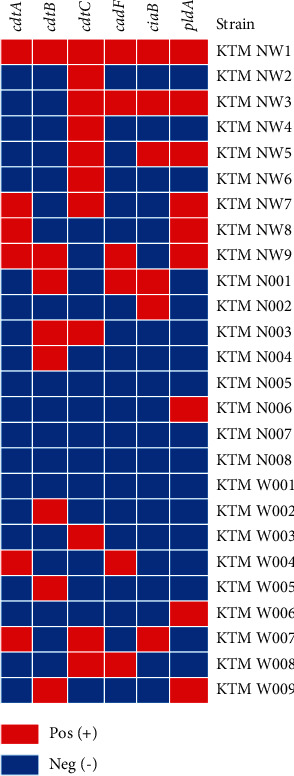
Distribution of the six virulence genes in *C. jejuni* isolates. Red colour represents the presence, and blue colour represents the absence of the virulence gene.

Many isolates carried *cdtC* (11/26; 42.3%), followed by *pldA* (9/26; 34.6%), then *cdtB* (8/26; 30.8%), *cdtA* (7/26; 26.9%), *cadF* (6/26; 23.1%), and *ciaB* (6/26; 23.1%). Multiple virulence genes were observed in seven (*n* = 7) isolates. The distribution of virulence genes in *C. jejuni* is presented on the heatmap (Figure 1).

### 3.2. Phylogenetic Analysis of *Campylobacter jejuni*

**Figure 2:**
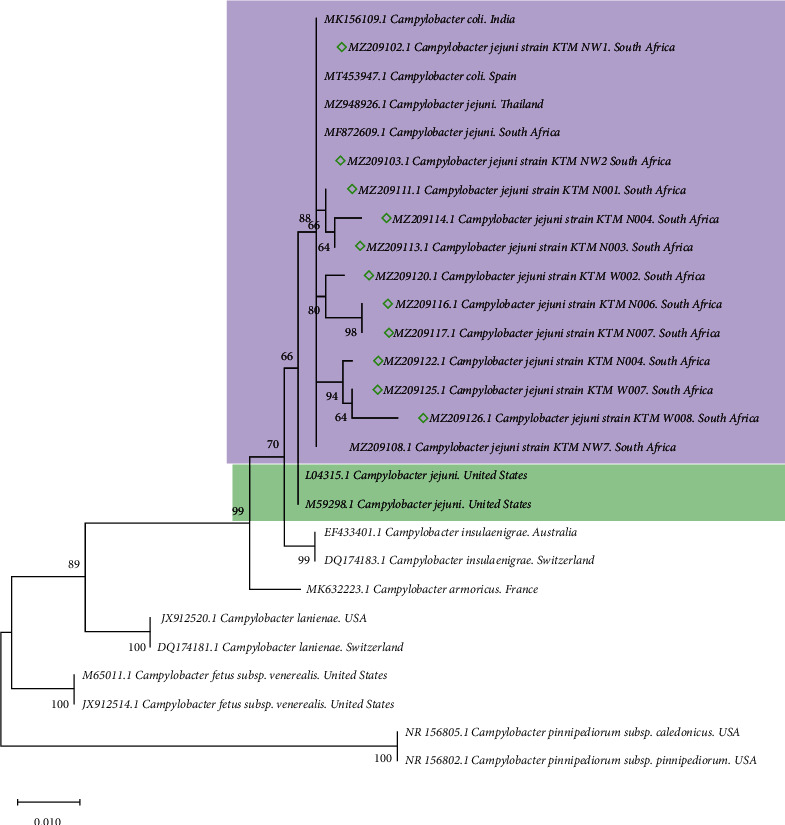
Phylogenetic tree of the *16S rRNA* gene constructed by using the maximum likelihood method and Kimura 2-parameter model among *Campylobacter* species. The node numbers represent the levels of bootstrap support based on 1000 replicates. The scale bar represents 0.010 substitutions per nucleotide position. All positions containing gaps and missing data were eliminated from the dataset (complete deletion option). The diamonds indicate *C. jejuni* isolates of the current study.

Based on the *16S rRNA* phylogenetic analysis, *C. jejuni* formed two well-supported monophyletic clades and were separated from other *Campylobacter* species. All the *C. jejuni* isolates from this study clustered in a single large monophyletic clade consisting of *C. jejuni* and *C. coli.* This clade represents a polytomy with both *campylobacter* species. Some of the isolates/strains from the current study formed poorly supported clades nested within this polytomy (Figure 2).

### 3.3. Antimicrobial Resistance Profile

Out of 26 tested isolates for the occurrence of AMR, a majority (96.1%; *n* = 25) of the isolates in this study showed resistance to nalidixic acid, followed by erythromycin (84.6%; *n* = 22), tetracycline (80.7%; *n* = 21), and ciprofloxacin (19.2%; *n* = 5) (Table 3).

### 3.4. Detection of Antibiotic Resistance Genes

**Figure 3:**
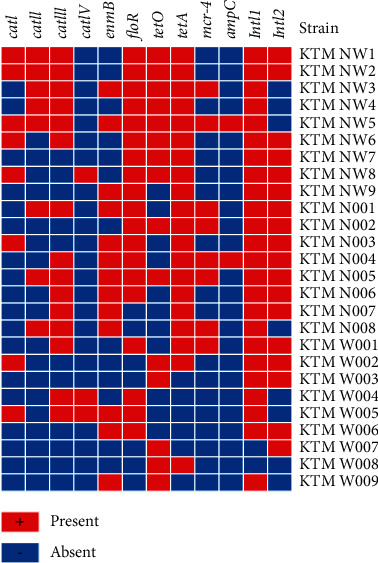
Antibiotic resistance profile of the *C. jejuni* strains using the 12 amplified antibiotic resistance genes in this study. The red colour represents the presence of antibiotic resistance genes, and blue represents the absence of antibiotic resistance genes.

Out of 19 genes investigated, only 63.2% (*n* = 12) were positively amplified (Table 3). Most of the isolates harbored chloramphenicol (*cat*I (*n* = 16; 84.2%), *cat*II (*n* = 15; 78.9%), *cat*III (*n* = 10; 52.6%), *cat*IV (*n* = 2; 10.5%), *flo*R (*n* = 10; 52.6%)), erythromycin (*erm*B (*n* = 14; 73.7%)), tetracycline (*tet*O (*n* = 13; 68.4%), *tet*A (*n* = 9; 47.4%)), colistin (*mcr-4* (*n* = 8; 42.1%)), and ampicillin (*amp*C (*n* = 2; 10.5%)) resistance genes. Thirty-one percent (*n* = 8) of the isolates were carrying more than two resistance genes, whereby most isolates carried class I and II integrons. About 92.3% (*n* = 24) isolates and 65.4% (*n* = 17) harbored class I and II integrons, respectively. The *mcr-1, mcr-2, mcr-3, mcr-5, tet*(X), *tet*(P), and *tet*(W) genes were not detected. A heatmap was generated to analyze the antibiotic resistance genes of *C. jejuni* used in this study (Figure 3). Eighty-one percent (*n* = 21) of the isolates were tetracycline (TET) resistant and carried *tet*O gene, 33% carried *tet*A gene, whilst 14.2% carried both *tet*O and *tet*A genes. The *erm*B gene, which confers erythromycin resistance, was present in the same *Salmonella* isolates (*n* = 12) that demonstrated phenotypic resistance. The occurrences of mismatch related to erythromycin (*erm*B) and tetracycline (*tet*A and *tet*O) were observed in eight and two isolates, respectively.

## 4. Discussion

This study was designed to determine the antibiotic resistance profiles and occurrence of virulence genes associated with pathogenesis mediated by many virulence factors [38] and the survival of *Campylobacter* spp. [10]. Six (*cdt*A, *cdt*B, *cdt*C, *cad*F, *pld*A, and *cia*B) virulence genes were assessed in this study including *Campylobacter* invasion antigens A, B, and C (*cdt*A, *cdt*B, and *cdt*C), the Cytolethal distending toxin (*cdt*) gene which encodes for a protein that releases cytotoxins that promote DNA damage [2, 10] and the inhibition of the cell cycle in G2 or M phase [15].

The presence of the *cdt* gene is linked with the severity of human campylobacteriosis. The *cdt*, which is encoded by three linked genes, namely, *cdt*A, *cdtB*, and *cdt*C, is one of the most well-studied virulence factors in *Campylobacter* spp. [10, 42]. In this study, the *cdtC* gene was the most prevalent gene (42.3%) followed by *cdtB* and *cdtA* with 30.8% and 26.9%, respectively. The detection of cytotoxicity genes (*cdt*A, *cdt*B, and *cdt*C) raises food safety concerns. Our results are in line with previous studies where *cdt*A, *cdt*B, and *cdt*C genes were detected in isolates from pigs and chickens [2, 10, 38]. In this study, 23.1% of the isolates harbored *Campylobacter* adhesion to the fibronectin (*cadF*) gene, which facilitates adherence to fibronectin in contact regions [15]. This was lower than the results obtained from previous studies where the *cadF* gene was detected in *Campylobacter* isolated from communal chicken, patriotic stool and water, human and cattle, children, and raw meat in South Africa [10, 20], Canada [43], Brazil [44], and South Africa [45], with the prevalence of 18.4%, 85.7%, 100%, 37.3%, and 85.7%, respectively.

The prevalence of the *pld*A gene was 34.6% which is high as compared to other studies conducted in South Africa, whereby this gene was detected at 7.4% [38]. This gene is responsible for adhesion and invasion, whereas the *cia*B gene which contributes to the invasion of epithelial cells [3, 46]. In this study, the *cia*B gene was detected in 23.1% of the isolates. Multiple virulence genes (*n* = 7) were observed in 12 isolates, and one isolate harbored all six virulence genes investigated in this study. These results are in line with the reports of Igwaran et al. and Han et al. [6, 47], where the isolates carried more than three virulence genes. The detection of these genes in these isolates indicates the pathogenic potential of the isolates [10]. They attach and invade the host epithelial cells [20], thus leading to detrimental effects on human health [3].

In our phylogenetic analysis, *Campylobacter* species isolated from avian/chicken hosts formed a well-supported monophyly with other closely related species of *Campylobacter* from a diverse range of vertebrate hosts. The clustering of *C. jejuni* and *C. coli* strains in one clade is similar to the findings reported in previous studies [48, 49]. The sequencing of the *16S rRNA* gene is commonly used to differentiate various bacterial species. However, given the highly conserved sequence similarity between the *16S rRNA* gene of *C. jejuni* and *C. coli,* the phylogenetic analysis results may demonstrate a closer relatedness than what is truly represented in the case. Furthermore, this underlines the importance of using the multigene target approach in discriminating and explaining the evolutionary history of these species globally, as highlighted by numerous authors [50, 51]. This approach could result in well-supported clades, and this polytomy of *C. jejuni* and *C. coli* could be resolved. Our two isolates were clustered together and both carried *Campylobacter* invasion antigens A, B, and C (*cdt*A, *cdt*B, *cdt*C, *cad*F, and *cia*B). Both isolates carried 70% of antibiotic resistance genes including class 1 and 2 integrons.

The use of antibiotics in the poultry industry has become a big concern globally due to the spread of antimicrobial resistance [52]. They are utilized in chicken farming companies for feed efficiency, growth promotion, and disease prevention [53–55]. Their continued use and misuse have resulted in the emergence of antibiotic-resistant *Campylobacter* [53, 54]. Between 1998 and 2011, ciprofloxacin resistance in clinical *C. jejuni* isolates from commercial chicken in South Africa increased from 1.4% to 79% [53].

Furthermore, between 1998 and 2011, tetracycline resistance in *C. jejuni* isolated from commercial poultry increased from 14.2% to 86% in South Africa, according to Basardien, [53]. Despite the fact that the therapeutic use of tetracycline in humans with campylobacteriosis has decreased in recent years [56], the high (80%) detection of the tetracycline resistance gene is not surprising in this study. Furthermore, the high resistance to nalidixic acid, erythromycin (macrolide that inhibits protein synthesis and translation) [57], and ciprofloxacin (fluoroquinolone that acts on bacterial DNA replication) [58] observed in this study raise more concerns to public health. This is because antibiotic resistance in *Campylobacter* spp., isolated from both humans and animals, has emerged as a major public health concern [11, 59].

The current study also investigated the occurrence of integrons (class I and II) and resistance gene patterns such as *cat*I, *cat*II, *cat*III, *cat*IV, *flo*R, *erm*B, *tet*(A), *tet*(O), *tet*(X), *tet*(P), *tet*(W), and *Amp*C of *C. jejuni* isolated from faeces of slaughter-age broiler chickens. Molecular detection of the antibiotic resistance gene disclosed the presence of 9 genes of which 2 were for tetracycline resistance (*tet*O (42.3%) and *tet*A (26.9%)). This finding is similar to previous findings of 92.3%, 83.1%, and 43.5% of *tet*O gene that was detected in pigs, poultry, and broiler chicken in South Africa, Iran, and China, respectively [6, 60, 61]. More specifically, about 81% of the isolates were tetracycline (TET) resistant and carried the *tet*O gene, 33% carried the *tet*A gene, and 14.2% carried both *tet*O and *tet*A genes. Tetracycline inhibits protein synthesis [58]. The *tet*O gene in *C. jejuni* can be found on the chromosome or, more commonly, on the plasmid pTet [18, 62, 63], and results in binding to the 30S subunit of ribosomes to inhibit protein synthesis [58, 64]. In *C. jejuni*, tetracycline resistance is encoded on a self-transmissible plasmid [65]. The alteration tetracycline ribosomal target and efflux are two known mechanisms of tetracycline resistance [18].

It is important to note that few (28%) isolates in this study carried the *amp*C gene encoding for ampicillin resistance which is a lower detection prevalence as compared to other reported studies with a 55% and 63% prevalence in South Africa from meat, milk, and water [6, 45]. Other ARGs detected included *cat*I, *cat*II, *cat*III, *cat*IV, *flo*R, and *amp*C and were 61.5%, 57.7%, 38.5%, 7.7%, 38.5%, and 8%, respectively. Colistin (polymyxin that acts in the destructuring bacterial cell membrane) has been screened by different studies phenotypically on *Campylobacter* spp. [64, 66–68]. However, the current study tested this antibiotic genotypically, resulting in 42.1% of the isolates carrying the *mcr-4* gene that encodes for colistin resistance. Some of the isolates were carrying more than two resistance genes. Our findings are also similar to a previous study in South Africa [39] where 33% of the isolates were carrying more than two resistance genes. Multidrug resistance genes discovered in *Campylobacter* isolates may limit treatment options for campylobacteriosis patients.

The class I integrons have been reported to harbor aminoglycoside resistance genes in *C. jejuni* [69]. Out of the 26 confirmed isolates, 88% carried the integrase gene (*IntI*1), a gene-encoding class 1 integrons. Similar findings concerning the predominance of class 1 integrons were reported previously by Chang et al. [70] and El-Aziz et al. [71], whereby 86% and 97% of the *Campylobacter* isolates from animals and humans carried the *intI*1 gene in Taiwan and Egypt, respectively.

## 5. Conclusion

This study revealed the occurrence of virulence and antibiotic resistance genes from *C. jejuni* isolated from faecal samples obtained from slaughter-age broiler chickens. The antimicrobial resistance tests indicated that *C. jejuni* isolates used in this study were resistant toward tetracycline, nalidixic acid, ciprofloxacin, and erythromycin and are further harboring antibiotic resistance genes (*cat*I, *cat*II, *cat*III, *cat*IV, *flo*R, *erm*B, *tet*O, *tet*A, *mcr-4*, and *amp*C) from different classes. The class I and II integrons were also detected in this study. According to our knowledge, this is the first study in South Africa to detect integrons II in *Campylobacter* spp. (*C. jejuni*). Due to the negative impact on human health of these findings on *Campylobacter*, cautious use of antibiotics in farming practices must be scaled up. Furthermore, we need to develop appropriate control measures to reduce the emergence of multidrug-resistant strains and to prevent the spread of strains carrying virulent genes.

## Figures and Tables

**Table 1:** Antibiotic resistance genes (ARGs), primers, and PCR conditions used in this study.

	Target gene	Primer	Primer sequence (5′ ⟶ 3′)	Conditions	Amplicon size (bp)	References
Tetracycline	*tet*(A)	TETA-FTETA-R	GCGCTNTATGCGTTGATGCAACAGCCCGTCAGGAAATT	94°C for 6 min (1x), 94°C for 30 s, 62°C for 30 s, 72°C for 60 s (30x), and 72°C for 6 min	387	[31]
	*tet*(O)	TETO-FTETO-R	ACGGARAGTTTATTGTATACCTGGCGTATCTATAATGTTGAC	94°C for 6 min (1x), 94°C for 30 s, 60°C for 30 s, 72°C for 60 s (30x), and 72°C for 6 min	171	[32]
	*tet*(X)	TETX-FTETX-R	CCGACACGGAAGTTGAAGAACCTTGGTGAGATGCCATTAGC	94°C for 6 min (1x), 94°C for 30 s, 60°C for 30 s, 72°C for 60 s (30x), and 72°C for 6 min	468	[32]
	*tet*(P)	TETP-FTETP-R	CTTGGATTGCGGAAGAAGAGATATGCCCATTTAACCACGC	94°C for 6 min (1x), 94°C for 30 s, 63°C for 30 s, 72°C for 60 s (30x), and 72°C for 6 min	676	[33]
	*tet*(W)	TETW-FTETW-R	GAGAGCCTGCTATATGCCAGCGGGCGTATCCACAATGTTAAC	94°C for 6 min (1x), 94°C for 30 s, 64°C for 30 s, 72°C for 60 s (30x), and 72°C for 6 min	168	[33]

Erythromycin	*erm*B	ERMB-FERMB-R	GCATTTAACGACGAAACTGGCTGACAATACTTGCTCATAAGTAATGGT	95°C for 2 min (1x), 95°C for 30 s, 60°C for 45 s, 72°C for 1 min (35x), and 72°C for 7 min	573	[34]

Colistin	*mcr-*1	mcr-1-Fmcr-1-R	TATCGCTATGTGCTAAAGCCTGCGTCTGCAGCCACTGGG	94°C for 5 min and 25 cycles, 94°C for 30 s, 56°C for 1 min, 72°C for 1 min, and 72°C for 5 min	1139	[35]
	*mcr-*2	mcr-2-Fmcr-2-R	TATCGCTATGTGCTAAAGCCTGAAAATACTGCGTGGCAGGTAGC		816	[35]
	*mcr-*3	mcr-3-Fmcr-3-R	CAATCGTTAGTTACACAATGATGAAGAACACATCTAGCAGGCCCTC		676	[35]
	*mcr-*4	mcr-4-Fmcr-4-R	ATCCTGCTGAAGCATTGATGGCGCGCAGTTTCACC		405	[35]
	*mcr-*5	mcr-5-Fmcr-5-R	GGTTGAGCGGCTATGAACGAATGTTGACGTCACTACGG		207	[35]

Ampicillin	*amp*C	AmpC FAmpC R	GTGACCAGATACTGGCCACATTACTGTAGCGCCTCGAGGA	95°C for 2 min, 35 cycles of 95°C for 30 s, 60°C for 45 s, 72°C for 1 min, and 72°C for 7 min	822	[36]

Chloramphenicol	catI	catI FcatI R	GGTGATATGGGATAGTGTTCCATCACATACTGCATGATG	1 min at 95°C, followed by 40 cycles of 15 s at 95°C, 30 s at 60°C, and 30 s at 72°C	349	[37]
	catII	catII FcatII R	GATTGACCTGAATACCTGGAACCATCACATACTGCATGATG		567	[37]
	catIII	catIII FCatIII R	CCATACTCATCCGATATTGACCATCACATACTGCATGATG		275	[37]
	catIV	CatIV F catIV R	CCGGTAAAGCGAAATTGTATCCATCACATACTGCATGATG		451	[37]
	*flo*R	FloR FFloR R	CGCCGTCATTCCTCACCTTCGATCACGGGCCACGCTGTGTC	1 min at 95°C, followed by 40 cycles of 15 s at 95°C, 30 s at 50°C, and 30 s at 72°C	215	[37]
Integrons	*IntI*1	*IntI*1-F*IntI*1-R	GCCTTGCTGTTCTTCTACGGGATGCCTGCTTGTTCTACGG	94°C for 5 min (1x); 30 s at 94°C, 30 s, 55–60°C, 2 min at 72°C (35x), and 5 min at 72°C	558	[35]
	*IntI*2	*IntI*2-F*IntI*2-R	CACGGATATGCGACAAAAAGGTGTAGCAAACGAGTGACGAAATG	94°C for 5 min (1x); 94°C for 1 min, 60°C for 1 min, 72°C for 2 min (32x), and 72°C for 10 min	740	[35]

**Table 2:** Primer sequences of virulence genes and PCR conditions used in this study.

Target gene	Primer	Primer sequence (5′ ⟶ 3′)	Conditions	Cycles	Size (bp)	References
*cdt*A	CDTA-FCDTA-R	CCTTGTGATGCAAGCAATC ACACTCCATTTGCTTTCTG	94°C for 15 min, 94°C for 1 min, 49°C for 1 min, and 72°C for 1 min, 72°C for 7 min	45	370	[39]

*cdt*B	CDTB-FCDTB-R	GTTAAAATCCCCTGCTATCAACCA GTTGGCACTTGGAATTTGCAAGGC	94°C for 15 min, 94°C for 1 min, 51°C for 1 min, and 72°C for 1 min, 72°C for 7 min	45	495	[39]

*cdt*C	CDTCFCDTCR	CGATGAGTTAAAACAAAAAGATA TTGGCATTATAGAAAATACAGTT	94°C for 15 min, 94°C for 1 min, 48°C for 1 min, and 72°C for 1 min, 72°C for 7 min	45	182	[39]

*cad*F	cadF-F2BcadF-R1B	TTGAAGGTAATTTAGATATGCTAATACCTAAAGTTGAAAC	95°C for 3 min, 94°C for 30 s, for 30 s, 43°C and 72°C for 1 min, 72°C for 5 min	45	400	[10]

*cia*B	CIAB-652CIAB R1159	TGCGAGATTTTTCGAGAATGTGCCCGCCTTAGAACTTACA	95°C for 3 min, 94°C for 30 s, for 30 s, 54°C and 72°C for 1 min, 72°C for 5 min	45	527	[38]

*pld*A	PLDA-FPLDA-R	AAGAGTGAGGCGAAATTCCAGCAAGATGGCAGGATTATCA	95°C for 3 min, 94°C for 30 s, for 30 s, 46°C and 72°C for 1 min, 72°C for 5 min	45	385	[38]

**Table 3:** Distribution of integrons, phenotypic, and genotypic antibiotic resistance in *C. jejuni* strains.

Samples ID	Strain	Accession number	Antibiotic class	Resistant genes pattern	Integrase	
					*IntI* I	*IntI* II
1	KTM NWI	MZ209102	NAL, TET, and ERY	*tet*A, *tet*O, *cat*I, *cat*II, *cat*III, and *flo*R	+	+
2	KTM NW2	MZ209103	TET and ERY	*tet*O, *cat*I, and *cat*II	+	+
3	KTM NW3	MZ209104	NAL, TET, and ERY	*mcr-4* and *erm*B,	+	−
4	KTM NW4	MZ209105	NAL, TET, and CIP	*tet*A, *cat*II, and *cat*III	+	−
5	KTM NW5	MZ209106	NAL, TET, and ERY	*mcr-4*, *amp*C, *erm*B, *tet*A, *tet*O, *cat*I, *cat*II, *cat*III, and *flo*R	+	−
6	KTM NW6	MZ209107	NAL, ERY, and CIP	*tet*A, *cat*I, and *cat*III	+	+
7	KTM NW7	MZ209108	NAL and TET	*tet*O and *flo*R	+	−
8	KTM NW8	MZ209109	NAL, TET and ERY	*tet*O, *cat*I, *cat*IV, and *flo*R	+	+
9	KTM NW9	MZ209110	NAL, TET, ERY, and CIP	*erm*B	+	+
10	KTM N001	MZ209111	NAL and TET	*mcr-4*, *erm*B, *tet*A, *cat*II, and *cat*III	+	+
11	KTM N002	MZ209112	NAL, TET, and ERY	*mcr-4* and *tet*O	+	+
12	KTM N003	MZ209113	NAL and ERY	*erm*B and *cat*I	+	+
13	KTM N004	MZ209114	NAL, TET, and ERY	*mcr-4*, *amp*C, *erm*B, and *cat*III	+	+
14	KTM N005	MZ209115	NAL, TET, and ERY	*mcr-4*, *erm*B, *tet*O, and *cat*II	+	+
15	KTM N006	MZ209116	NAL and ERY	*erm*B and *flo*R	+	+
16	KTM N007	MZ209117	NAL, TET, and ERY	*erm*B	+	+
17	KTM N008	MZ209118	NAL, TET, and ERY	*mcr-4*, *erm*B, *tet*A, and *cat*II	+	−
18	KTM W001	MZ209119	NAL, TET, and ERY	*mcr-4*, *tet*A, *cat*III, and *flo*R	+	+
19	KTM W002	MZ209120	NAL, TET, and ERY	*tet*O, *tet*A, and *cat*I	+	+
20	KTM W003	MZ209121	NAL, TET, and ERY	*tet*O	+	+
21	KTM W004	MZ209122	NAL and ERY	*erm*B, *tet*O, *cat*III, and *cat*IV	+	−
22	KTM W005	MZ209123	NAL, TET, ERY, and CIP	*erm*B, *cat*I, *cat*III, and *flo*R	+	−
23	KTM W006	MZ209124	NAL and ERY	*erm*B and *flo*R	+	+
24	KTM W007	MZ209124	NAL, TET, and ERY	*tet*O	−	+
25	KTM W008	MZ209126	NAL, TET, ERY, and CIP	*tet*A and *tet*O	−	−
26	KTM W009	MZ209127	NAL and TET	*erm*B and *tet*O	+	−

## Data Availability

The datasets generated and analyzed will be available upon request to the corresponding author.
